# Bridging the gap between current practice recommendations in national guidelines - a qualitative study of mental health services

**DOI:** 10.1186/1472-6963-14-S2-P94

**Published:** 2014-07-07

**Authors:** Monica Stolt Pedersen, Anne Landheim, Lars Lien

**Affiliations:** 1 National Centre for Dual Diagnosis, Innlandet Hospital Trust, Ottestad, Norway; 2 Faculty of Medicine, University of Oslo, Oslo, Norway; 3 Norwegian Centre for Addiction Research, University of Oslo, Oslo, Norway

## Background

“National guideline for assessment, treatment and social rehabilitation of persons with concurrent substance use disorders and mental disorders”, launched March 2012, is aimed at a wide range of health services. It holds a separate chapter on implementation.

The National Centre for Dual Diagnosis was commissioned by the Norwegian Directorate of Health to develop a plan for implementation of the Guideline. It contains tools to strengthen management, clinicians and consumers. A clinical audit tool was made to measure research-practice-gap. The clinical audit is the start-point of the implementation-plan, followed by an action schema. The implementation-process is as follows:

- Identify today’s practice assessed against recommendations in the guideline, doing a clinical audit

- Identify areas of improvement based on the clinical audit

a) choose a goal for improvement

b) select initiatives based on goals

c) allocate responsibility

d) describe progress

- Implementation phase

- Evaluate by a new clinical audit and summarize experiences

This project aims to understand the process of using clinical audit as a basis for making choices aimed at clinical improvement in district psychiatric clinics. The objectives of the study are to describe and explore the implementation-process from the use of clinical audit to change in practice.

## Materials and methods

Three different methods will be used, all qualitative, to explore the process: 1) observation of meetings, minutes of meetings etc. from the presentation of results from the clinical audit to a fully described action schema, 2) focus-group interview with participants from each of the included units after second clinical audit, and 3) individual interviews with head of the units after second clinical audit. There is a desire to open up for experiences, perceptions, attitudes and narratives.

The setting is four units at a district psychiatric clinic, one outpatient clinic, two inpatient units, and one psychiatric outpatient emergency team.

## Results

First observations are done and analysis pending. Preliminary results from the observation part of the study may be presented in July.

